# Magnesium Oxide Reduces Anxiety-like Behavior in Mice by Inhibiting Sulfate-Reducing Bacteria

**DOI:** 10.3390/microorganisms12071429

**Published:** 2024-07-14

**Authors:** Cristina N. Coffman, Amanda Carroll-Portillo, Joe Alcock, Sudha B. Singh, Kellin Rumsey, Cody A. Braun, Bingye Xue, Henry C. Lin

**Affiliations:** 1 Biomedical Research Institute of New Mexico, Albuquerque, NM 87108, USA; ccoffman1@unm.edu (C.N.C.); sudha.singh@va.gov (S.B.S.); cody.braun1@va.gov (C.A.B.); bingye.xue@va.gov (B.X.); 2 New Mexico VA Health Care System, Albuquerque, NM 87108, USA; acarrol1@unm.edu; 3 Division of Gastroenterology and Hepatology, University of New Mexico, Albuquerque, NM 87131, USA; 4 Emergency Medicine, University of New Mexico, Albuquerque, NM 87131, USA; joalcock@salud.unm.edu; 5 Statistical Sciences, Los Alamos National Laboratory, Los Alamos, NM 87545, USA; knrumsey@unm.edu

**Keywords:** sulfate-reducing bacteria (SRB), *Desulfovibrio*, Deferribacterota, dysbiosis, hydrogen sulfide (H_2_S)

## Abstract

The gut microbiota–brain axis allows for bidirectional communication between the microbes in our gastrointestinal (GI) tract and the central nervous system. Psychological stress has been known to disrupt the gut microbiome (dysbiosis) leading to anxiety-like behavior. Pathogens administered into the gut have been reported to cause anxiety. Whether commensal bacteria affect the gut–brain axis is not well understood. In this study, we examined the impact of a commensal sulfate-reducing bacteria (SRB) and its metabolite, hydrogen sulfide (H_2_S), on anxiety-like behavior. We found that mice gavaged with SRB had increased anxiety-like behavior as measured by the open field test. We also tested the effects of magnesium oxide (MgO) on SRB growth both in vitro and in vivo using a water avoidance stress (WAS) model. We found that MgO inhibited SRB growth and H_2_S production in a dose-dependent fashion. Mice that underwent psychological stress using the WAS model were observed to have an overgrowth (bloom) of SRB (Deferribacterota) and increased anxiety-like behavior. However, WAS-induced overgrowth of SRB and anxiety-like behavioral effects were attenuated in animals fed a MgO-enriched diet. These findings supported a potential MgO-reversible relationship between WAS-induced SRB blooms and anxiety-like behavior.

## 1. Introduction

The gut microbiota–brain axis is a complex bidirectional signaling pathway between the microbial community in the gut and the brain. The brain and the microbes in our gastrointestinal tract communicate via afferent, efferent, neuroendocrine, and immune pathways [1,2]. Psychological stress, whether chronic or acute, is a powerful challenge that also has a well-documented impact on the gastrointestinal tract. For example, stress has been shown to exacerbate gastrointestinal disease symptoms in patients with inflammatory bowel disease (IBD), irritable bowel syndrome (IBS), and Crohn’s disease [3,4,5]. A contributing role of the gut microbiome is suggested by studies showing that exposure to psychological stress or neuroendocrine hormones linked to stress alters the composition of the gut microbiota (dysbiosis) [6,7,8]. These stress-related changes in the microbiome have been shown to result in changes in behavior. Mice exposed to unpredictable mild chronic stress showed increased anxiety-like behavior and depressive-like behavior [9,10]. These behavioral changes can be transferred by fecal transplantation from stressed mice to unstressed mice, indicating a causal relationship between bacteria and behavior [11]. Additionally, behavioral changes associated with stress in rats have been observed even 1 month after exposure to stress [12]. This indicated the possibility that sustained behavior changes after short-term stress exposure could be due to a perturbed microbiome or dysbiosis.

Exposure to pathogens has a similar impact on behavior. Specifically, treating mice with *Citrobacter rodentium* via gavage triggered anxiety-like behavior compared with controls [13]. The behavioral changes were postulated to be the result of bacterial metabolites impacting the neural pathway rather than an effect of immune activation [14]. As most microbiome studies focus on large microbial compositional changes, the role of rare resident gut bacteria is poorly understood. One group of resident gut bacteria of interest is sulfate-reducing bacteria (SRB), a group of rare commensal Gram-negative bacteria that are normally present in low concentrations but increase in number (overgrowth or bloom) in a variety of disease states associated with gut dysbiosis [15,16]. Additionally, the SRB *Desulfovibrio* represents the 20 most abundant genera in humans, with an emerging role reported in host health and disease [17]. We showed, previously, that administering SRB via oral gavage acutely impaired working memory in mice [18]. This indicates that SRB could rapidly impact the gut–brain axis, perhaps via its metabolic by products.

SRB generates hydrogen sulfide (H_2_S) as a metabolic by product using hydrogen produced by microbial fermentation as the substrate. H_2_S is also produced by mammalian cells and functions as a gasotransmitter that can play a role in regulating a variety of physiologic processes. However, at higher concentrations, this gas can be toxic and even lethal. Individuals accidentally exposed to high concentrations of H_2_S have reported adverse effects on cognition and behavior, including anxiety and depression, and damage to lung, heart, vascular, and brain tissues [19,20]. Our previous work showed that the stress hormone norepinephrine increased the growth of SRB and the production of H_2_S [21]. This result demonstrated the connection between stress and these bacteria and its metabolism.

Supplemental magnesium is widely prescribed for conditions as varied as constipation, muscle cramps, and bone health [22,23,24]. It is generally regarded as safe, especially in individuals with normal kidney function. The intake of magnesium has been associated with reduced anxiety in humans [25,26,27]. Similarly, in mice fed a magnesium-deficient diet, anxiety-like behavior was observed, along with a change in the composition of the gut microbiome [28]. Magnesium may have a therapeutic effect, as supplementation with magnesium lowered the cortisol level, a marker of stress [29]. It is not known, however, whether magnesium is effective in treating anxiety and SRB overgrowth. In this study, we tested the hypothesis that water avoidance stress (WAS) may induce an overgrowth of SRB and increase anxiety-like behavior in mice in a magnesium-reversible fashion.

## 2. Materials and Methods

### 2.1. Experimental Outline

**Figure 1:**
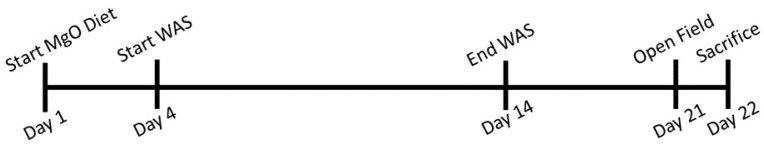
Timeline of WAS experiment. Mice were placed on their respective diets three days prior to WAS and were kept on the diet until the end of the experiment. WAS began on day 4 of the experiment and was conducted for 1 h a day for 10 consecutive days. Anxiety-like behavior was assessed by an open field test on day 21 and sacrificed followed on day 22.

The first in vivo experiment tested the effects of SRB on anxiety-like behavior in mice. Mice were gavaged with SRBs in the form of *Desulfovibrio vulgaris* and anxiety-like behavior was assessed using the open field test 2 h post gavage. We conducted an in vitro experiment, looking for a potential treatment of SRB overgrowth, and examined the effects of magnesium oxide (MgO), a poorly absorbable formulation active in the gut lumen, on SRB growth and H_2_S production. We then conducted an in vivo experiment using a mouse model to determine the impact of psychological stress (water avoidance stress, WAS) on the SRB overgrowth and behavior (Figure 1). Mice were subjected to water avoidance stress (WAS) for 1 h a day for 10 consecutive days with vs. without a MgO enriched diet. Open field was used to assess anxiety-like behavior, and 16S sequencing on small intestinal tissue was used to determine potential SRB blooms. Experimental protocols were approved by the Institutional Animal Care and Use Committee of the New Mexico VA Health Care System, following guidelines provided by the Guide for the Care and Use of Animals of the National Research Council of the National Academies [30].

### 2.2. Effects of SRB Gavage on Anxiety-like Behavior

Sixteen five-week-old female C57Bl/6 (15–20 g) mice from Charles River Laboratories (Wilmington, DE, USA) were housed in groups of four in polypropylene cages on a twelve hour light/dark cycle. Mice were randomly divided into 2 groups of 8. *Desulfovibrio vulgaris* subsp. *vulgaris* was purchased from ATCC (29579; Manassas, VA, USA) and was used as our representative SRB. We chose this strain of bacteria because it represents the most common genus of SRB in the gut [31]. Mice were gavaged with 100 µL of SRB (1 × 10^9^ cells total) resuspended in lactulose solution (33 mg/mL) from Cumberland Pharmaceuticals (Kristalose; Nashville, TN, USA). We used lactulose as a fermentable substrate for hydrogen-producing gut bacteria. Control mice were gavaged with 100 µL lactulose at 33 mg/mL resuspended in PBS. SRB concentrations were determined based on previous piloting, which demonstrated observable behavioral effects using a dose of 1 × 10^9^ cells total. The mice were monitored after the SRB gavage and no adverse side effects were observed.

### 2.3. Open Field Test

Mice were assessed for anxiety-like behavior using an open field arena (44.5 cm length × 44.5 cm width × 21.5 cm height). Mice were acclimated to the behavior room in their home cages an hour before testing started. The arena was illuminated with an 800-lumen bulb and divided into a 6 cm grid. Three zones—edge, middle, and center—were monitored by a video camera. Animals were released in the center of the arena and monitored for a period of 5 min. We chose this time point because previous piloting indicated that 5 min was the optimal time to observe differences in anxiety-like behavior between groups. Mice who received SRB via oral gavage were assessed for anxiety-like behavior 2 h post gavage; the outcomes included latency to first enter the edge zone (shorter latency corresponds to anxiety-like behavior), number of line crossings into the edge zone (more crossings correspond to anxiety-like behavior), latency to first exit the center zone (shorter latency corresponds to anxiety-like behavior), and total time spent in the center zone (shorter time corresponds to anxiety-like behavior). For mice that underwent WAS, anxiety-like behavior was assessed on day 7 post WAS. Previous piloting showed that day 7 post WAS was the earliest timepoint for detecting significant change in gut microbial composition consistent with gut dysbiosis and SRB blooms. The outcomes included latency to first exit edge zone and latency to first entering the center zone. Measurements were quantified using ANY-maze video tracking software (Version 6.0; Stoelting Co., Wood Dale, IL, USA).

### 2.4. In Vitro: Effects of MgO on SRB Growth and H_2_S Production

We wanted to test the effects of MgO on SRB growth to test for a potential treatment. *Desulfovibrio vulgaris* was used as the represented SRB. We examined whether MgO had direct effects on SRB growth and H_2_S production. Bacteria was grown in 5 mL of modified Postgate’s organic liquid media in anaerobic Hungate tubes (10.56 mM Na_2_SO_4_, 13.29 mM MgSO_4_, 4.12 mM L-Cysteine, 0.4% sodium lactate (60% syrup), 0.4% yeast extract, and 0.5% tryptone), with a starting concentration of 1.0 × 10^6^ cells/mL. SRB was grown for 24 h at 37 °C, with MgO added to the media to achieve varying concentrations of 0.25, 0.5, 1.0, 1.5, and 2.0 mg/mL. Bacterial growth was measured 24 h post inoculation using the QuantomTx Microbial Cell Counter (Logos Biosystems, Anyang-si, Republic of Korea). The Quantom Total Cell Staining Kit was used to stain cells according to the manufacturer’s protocol. H_2_S was measured using a gas chromatography (OralChroma, Nissha FIS Inc., Osaka, Japan) following methods used in previous studies [18,21,32,33]. Media alone was used as a blank and these readings were subtracted from the bacterial gas readings to account for any background H_2_S. Measurements of H_2_S concentration in parts per billion (pbb) within the airtight chamber of the Hungate tubes were recorded at 24 h.

### 2.5. In Vivo: Effect of MgO on WAS-Induced Behavior

Forty five-week-old female C57Bl/6 (15–20 g) mice from Charles River Laboratories (Wilmington, DE, USA) were housed in groups of four in polypropylene cages on a twelve hour light/dark cycle and kept on a standard rodent diet during the one-week acclimation period (Teklab Global Rodent Diets, 2920X). Mice were randomly assigned to 1 of 5 groups (n of 8 each) consisting of control (MgO at 0 mg/Kg_bw_), MgO at 100 mg/Kg_bw_, WAS, WAS + MgO at 10 mg/kg_bw_, and WAS + MgO at 100 mg/kg_bw_. Behavior was assessed on day 7 post WAS and mice were euthanized on day 8 post WAS. One data point from the control and one data point from the WAS + 100 mg/kg_bw_ group were removed due to recording errors.

### 2.6. Magnesium-Supplemented Diet

The recommended daily dose of magnesium is 7.5 mg/kg_bw_ in healthy adult males [34]. Supplemental dosing in human studies can range from recommended daily intake to higher dosing (60 mg/kg_bw_) [35]. The dosing of rodents with magnesium can vary widely. We chose our dosing based on previous studies using 100 mg/kg_bw_ of magnesium in rats as this is considered a mid-range dose for magnesium supplementation in rodent studies [36,37]. We also chose a 10 mg/kg_bw_ dose to determine whether a dose dependent response would occur. Magnesium oxide was used as the source of magnesium in this experiment as it is poorly absorbed and more likely to target gut bacteria (magnesium oxide, Bulk Supplements, Nevada) and was added to the standard rodent diet (Teklab Global Rodent Diet: 2920X, Inotiv, West Lafayette, IN, USA) in doses of 10 mg/Kg_bw_ and 100 mg/Kg_bw_. Calculations were performed assuming each mouse consumed 3 g of food per day at an average weight of 20 g [38,39]. Food was consumed ad libitum. Mice were started on their respective diets 3 days prior to WAS treatment and kept on the diet until euthanasia (Figure 1).

### 2.7. Water Avoidance Stress

Mice underwent 10 consecutive days of WAS for 1 h each day. The time and duration of stress was determined by previous studies [40,41]. All stress sessions were performed at a similar time of the day (between 8:00 and 10:00 a.m.). The test apparatus consisted of a clear plastic container (30 cm length × 30 cm width × 26 cm height) with a clear cylindrical plastic pedestal (7.62 cm height × 3.81 cm width) attached to the base of the container. The animals were able to stand upright and stable on the top of the pedestal. The container was filled with room temperature water (~24 °C) to within 1 cm of the top of the pedestal. Control mice were left in their cages untouched during this time. The mice were monitored throughout the duration of the experiment for signs of discomfort or adverse effects from the MgO-enriched diet and WAS treatment (diarrhea, weight loss, excessive grooming, etc.) and we observed no adverse side effects.

### 2.8. Effects of MgO on SRB Density in the Small Intestine

As sulfate-reducing bacteria comprise only a small percentage of the gut microbiome [42], we decided to use sequencing analysis to help quantify these rare members of the bacterial community. Small intestinal tissue was collected and preserved in RNA/DNA Shield (Zymo Research, Irvine, CA, USA) for sequencing. Samples were extracted and processed by the University of Minnesota Genomic Center using the QIAgen DNeasy kit following the manufacturer’s protocols (Qiagen, Hilden, Germany), and 16S rRNA gene sequencing targeting the V3-V6 region was performed on the mucosa-associated microbiome of the mid-jejunum to test for the presence of a bloom of sulfate-reducing bacteria. Sequencing was performed using the MiSeq platform (Illumina, San Diego, CA, USA) and protocols were used according to the manufacturer with some modifications [43]. Bioinformatic analysis was conducted by CD Genomics (Shirley, New York, NY, USA).

### 2.9. Statistical Analyses

In total, there were nine independent variables explored in this study. For assessing the effects of SRB gavage on anxiety-like behavior, four variables were explored as follows: (i) latency to first enter the edge, (ii) number of line crossings, (iii) latency to first exit the center, and (iv) total time spent in the center. For each of these, the approximate normality of both the control and SRB group was positively determined with a 95% QQ-plot envelope. Confidence intervals (90%) were constructed using standard t-procedures. Welch’s *t*-test was used to compare the mean between control and SRB groups.

To analyze the effects of MgO on SRB growth and H_2_S production, the following two independent variables were explored: H_2_S concentration (ppb) and SRB growth (cells/mL). These variables were both heavily right-skewed and the H_2_S production variable had several zero values due to limitations of the OralChorma not being sensitive enough to read H_2_S levels in samples that contain low concentrations of bacteria. Using a 90% QQ-plot envelope, it was determined that a log-normal model was appropriate for SRB growth, and 90% confidence intervals were constructed using Cox’s method [44]. Similarly, it was determined that a zero-inflated log-normal model was appropriate for H_2_S production [44] and 90% confidence intervals were constructed via the parametric bootstrap algorithm with maximum likelihood estimation [45]. To test the relationship between these variables and MgO dosage level (administered at 6 levels between 0 mg/mL and 2 mg/mL), we fit a simple linear regression model after applying transformations to the data (log for SRB growth and shifted-log for H_2_S Production). Analysis of the residuals indicated that the model was robust to the standard assumptions.

Next, the (in vivo) effects of MgO on WAS-induced behavior was studied, with two independent variables: latency to first exit the edge zone and latency to first enter the center zone. The explanatory variables were (i) the presence of WAS and (ii) the dosage level of MgO (administered at 3 levels between 0 mg/Kg_bw_ and 100 mg/Kg_bw_). Across all treatment groups, the normality assumption was approximately satisfied for both independent variables, using a QQ-plot envelope test. Two groups for the second independent variable showed slight deviations from normality, but a further analysis of the sampling distribution of the sample mean (using a non-parametric bootstrap [46]) indicated that the results should be robust, or at least conservative, to this mild deviation. Thus, 90% confidence intervals were constructed using standard t-procedures. All treatment groups were compared with the control group using Welch’s *t*-test with a Sidak correction to account for multiple comparisons.

Finally, there was a single independent variable for describing the effects of MgO on SRB density in the small intestine (measured in % composition). For analysis, a shifted-log transformation was applied to the response variable and 90% confidence intervals were obtained using Cox’s method [43]. Using the presence of WAS and the dosage level of MgO as predictor variables, a simple linear model was fit to the data, producing an F-test and *t*-tests for each model coefficient. Analysis of the residuals indicated that all assumptions were met. SRB values were represented as percentage and were multiplied by 1000 for better readability. Mean and lower/upper bounds of a 90% CI were reported, and a two-sided *p*-value (with a Sidak correction) for comparing the mean of each group with the control group mean.

All statistical analyses were performed using R software (R Core Team, 2024) and all statistical figures were made with the ggplot2 package in R [47]. In accordance with the ASA guidelines [48], we did not adhere to a fixed significance level but rather presented effect sizes, confidence intervals, and *p*-values to allow for a more nuanced presentation of the results.

## 3. Results

### 3.1. Effects of SRB Gavage on Anxiety-like Behavior

**Figure 2:**
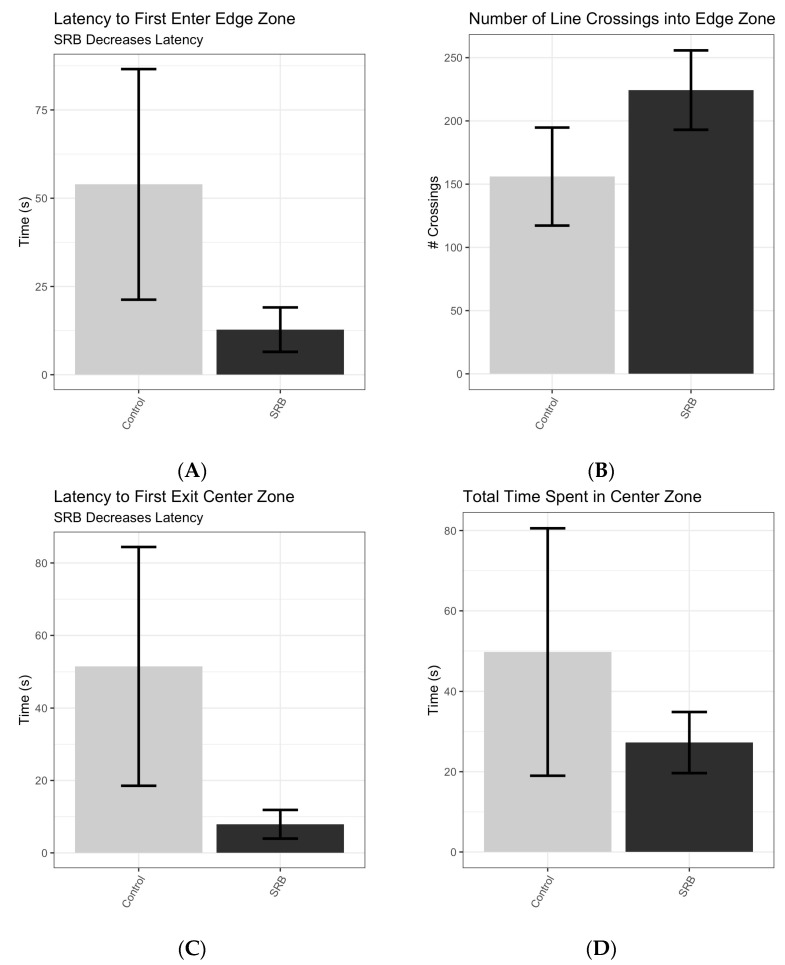
SRB increase anxiety-like behavior in mice. Mice were gavaged with SRB in the form of *Desulfovibrio vulgaris* resuspended in lactulose solution (33 mg/mL) at 1 × 10^9^ cells total. Control mice were gavaged with 100 µL lactulose at 33 mg/mL resuspended in PBS. Mice were placed in an open field arena and behavior was recorded for 5 min. Latency to first enter edge zone(s) (**A**), number (#) of line crossings (**B**), latency to first exit center zone(s) (**C**), and total time spent in center zone(s) (**D**) were measured. An unpaired *T*-test was used to compare groups. The mean values are presented, and the error bars represent 90% confidence intervals for the mean.

On average, mice that were gavaged with SRB entered the edge zone more than four times as quickly as control animals, taking just 12.78 (±4.44) seconds compared with 53.91 (±22.68) seconds for the control group (*p* = 0.122; Figure 2A). Additionally, SRB-gavaged mice (224 ± 16.56 crossings) crossed into the edge zone significantly more than the control mice (156 ± 20.45 crossings *p* = 0.021; Figure 2B). We also measured the time it took each mouse to leave the center zone after first entering. Mice who were gavaged with SRB stayed in the center zone significantly less (7.9 ± 2.0 s) compared with the control group (51.48 ± 17.39 s, *p* = 0.041) and were represented by latency to first exit the center zone (Figure 2C). Additionally, SRB-gavaged mice spent half the amount of time (27.26 ± 4.01 s) as the control mice in the center zone (49.78 ± 16.25 s, *p* = 0.216; Figure 2D).

### 3.2. Effects of MgO on SRB Growth and H_2_S Production

**Figure 3:**
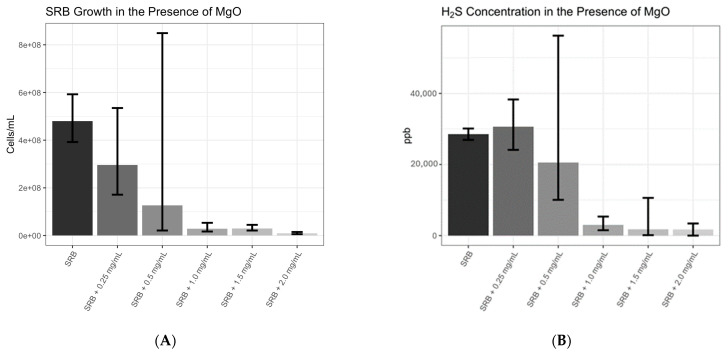
MgO inhibits growth and H_2_S production of *SRB.* Bacteria was grown with varying doses of MgO (0–2.0 mg/mL) in liquid media at 37° for 24 h (n = 3). Total counts were obtained and represented as cells per ml (**A**). To account for the heavy right skew of the response variable, the analysis was conducted using a shifted log-transformation of the data and 90% confidence intervals were constructed using Cox’s method. Hydrogen sulfide production of SRB grown for 24 h with varying concentrations of MgO as represented as parts per billion (ppb) (**B**). The 90% confidence intervals were constructed using a parametric bootstrap procedure based on a zero-inflated log-normal model where the parameters were estimated using maximum likelihood. The relationship between MgO dose level and the response (SRB growth and H_2_S concentration) was assessed with an F-test based on a simple linear model. Data are presented as Mean ± SEM.

We tested the effect of MgO on the growth of *Desulfovibrio* spp. at varying concentrations (0, 0.25, 0.5, 1.0, 1.5, and 2.0 mg/mL). We found evidence of a strong relationship between SRB growth and exposure to MgO (F-test: *p* = 3.8 × 10^−11^), as shown in Figure 3A. A simple log-linear model clearly demonstrated that SRB growth was significantly suppressed by increased exposure to MgO (slope coefficient = −1.87, *p* = 3.8 × 10^−11^). Similarly, Figure 3B demonstrates that H_2_S concentration declined with increasing exposure to MgO (F-test: *p* = 2.9 × 10^−5^) based on a simple log-linear model with a small offset chosen to satisfy the statistical assumption of normality (slope coefficient = −6.99, *p* = 2.9 × 10^−5^).

### 3.3. In Vivo: Effect of MgO on WAS-Induced Anxiety-like Behavior

Based on a simple linear model using MgO dosage and the presence of WAS as predictors, an F-test suggested a possible relationship between these predictors and the latency to first exit the edge zone, though the evidence was not particularly strong (*p* = 0.097).

**Figure 4:**
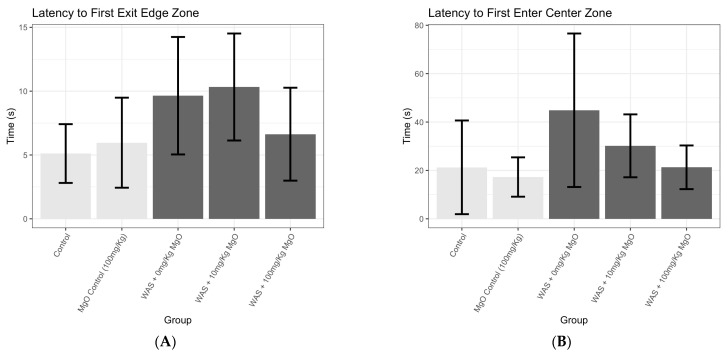
Magnesium oxide at high-dose attenuated stress induced anxiety-like behavior. Mice were placed in an open field arena and behavior was recorded for 5 min. Latency to first exit edge zone(s) (**A**) and latency to first enter center zone(s) (**B**) were measured. The mean latency times are presented, and the error bars represent 90% confidence intervals for the mean.

As seen in Figure 4A and in Table 1, at a fixed MgO dosage level, the WAS-treated mice generally took longer to exit the edge zone. However, in animals fed the MgO-enriched diet (WAS + 100 mg/kg_bw_), the anxiety-like behavior was similar to the controls. Using a Sidak correction to adjust for multiple comparisons, there was some limited evidence to suggest that the WAS + 0 mg/kg_bw_ group (4.53 s difference, *p* = 0.222) and the WAS + 10 mg/kg_bw_ group (5.21 s difference, *p* = 0.0997) differed from the controls. In the high-dose group (WAS + 100 mg/kg_bw_), however, there was no evidence to suggest a meaningful difference (1.52 s difference, *p* = 0.881) from the controls, suggesting a potential effect of MgO at 100 mg/kg_bw_ inhibiting WAS-induced anxiety-like behavior in mice (Figure 4A; Table 1). As seen by Figure 4B and Table 2, similar results were found for the latency time to first enter the center zone (F-test: *p* = 0.087). While WAS generally increased anxiety-like behavior (44.87 ± 12.95 s), mice fed a magnesium-enriched diet (WAS + 100 mg/kg_bw_; 21.3 ± 3.5 s) showed anxiety-like behavior no greater than the controls (21.28 ± 7.91 s). Overall, the data confirmed the hypothesis that, while WAS increases anxiety-like behavior in mice, a MgO-enriched diet at 100 mg/kg_bw_ dose may reduce this effect.

### 3.4. Effects of MgO on SRB Density in the Small Intestine

**Figure 5:**
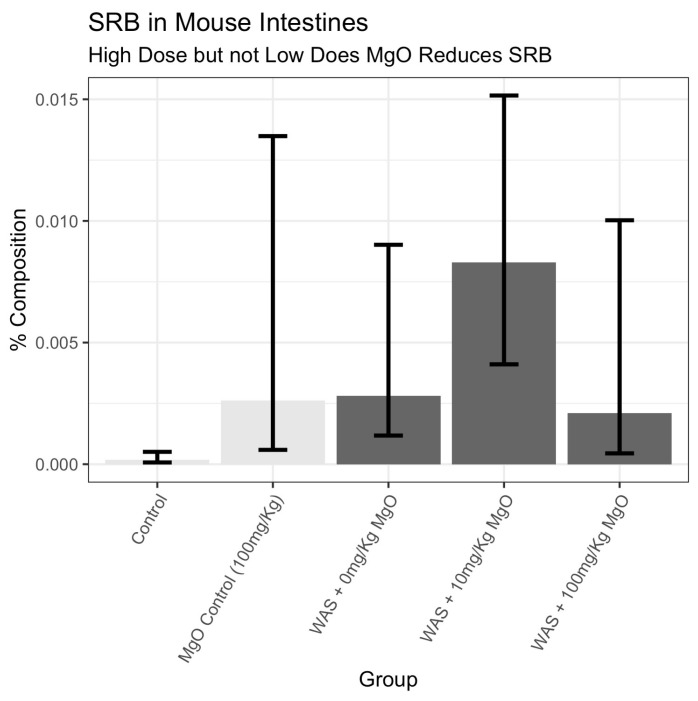
High-dose, but not low-dose, MgO suppressed SRB in the mouse intestine. The graph depicts the percent composition of SRB in the form of Deferribacterota in the jejunum of control vs. WAS-treated (stress) mice with and without a MgO-enriched diet. The mean % composition for each group is presented, and the error bars represent 90% confidence intervals for the mean.

Sequencing analysis of the mucosa-associated microbiome of the jejunum revealed that mice who underwent WAS treatment had substantial increases in SRB, as seen by changes in Deferribacterota concentrations (Figure 5). There were increases in SRB between all groups compared with the control group, except the for the WAS + 100 mg/kg_bw_ group, which was similar to the control group, demonstrating that MgO at 100 mg/kg_bw_ dose was effective. The largest difference, by far, was found between the control group and the WAS-treated mice with a low dose (WAS + 10 mg/kg_bw_) or no dose of MgO in the diet (WAS). This suggests that WAS increased SRB in the animals; however, a high MgO-enriched diet at 100 mg/kg_bw_ may counteract this effect.

In another analysis, a simple linear model was fitted to the transformed Deferribacterota data using MgO dosage level and WAS presence as predictors (F-test: *p* = 3.5 × 10^−5^). In this model, the coefficient for WAS presence was positive and highly significant, as expected (coefficient = 3.24, *p* = 2 × 10^−6^), indicating that it was related to an increase in SRB. The coefficient for MgO was small, but positive (coefficient = 0.020, *p* = 0.0045), but the coefficient corresponding to the interaction was negative and considerably larger (coefficient = −0.034, *p* = 0.0004). As stated before, these data suggested that WAS increased SRB concentrations in the small intestines of mice. Additionally, SRB increased slightly with MgO dosage; however, when WAS was combined with a high dose of MgO (100 mg/kg_bw_), this reduced the increase in SRB concentrations drastically close to control levels (Figure 5; Table 3).

## 4. Discussion

During dysbiosis, a change in the composition of the microbial community occurs, leading to an imbalance of different members. It is well reported that dysbiosis may lead to an increase in number of pathogenic bacteria due to a loss of colonization resistance [6]. Bailey and Coe [49] showed that the stress of maternal separation in rhesus monkeys caused a loss of protective lactobacilli that opened a niche for colonization and blooms of the pathogenic bacteria *Shigella* and *Campylobacter.* For example, when mice are gavaged with a single pathogen, such as *Citrobacter rodentium*, this can trigger anxiety-like behavior [13]. Similarly, when SRB concentrations increase during dysbiosis, they can have pathogenic effects [31,50]. In this study, we found that mice gavaged with SRB had increased anxiety-like behavior compared with control mice (Figure 2A–D). SRB and hydrogen sulfide increases have been observed in ulcerative colitis patients [16,51]. Additionally, anxiety is a well-reported feature of these conditions and stress triggers their exacerbation [5,14], further supporting the need for preventative and treatment methods of SRB blooms. 

Magnesium deficiency has been linked with reduced microbial diversity, which has been associated with increases in anxiety-like [28] and depression-like behaviors [52]. Our findings raised the following interesting question: could the beneficial effect of an adequate magnesium intake be explained by its effect in suppressing the number of SRB in the gut? Magnesium oxide has been reported to have antibacterial characteristics through the generation of reactive oxygen species (ROS) such as superoxide (O_2_^−^) [53]. Additionally, magnesium peroxide has been reported to inhibit growth of a mixed SRB population in a laboratory setting [54]. While magnesium oxide was shown to inhibit the growth of bacterial pathogens, including *Escherichia coli* and *Staphylococcus aureus*, the primary reason for this effect was through the release of ROS [53]. We showed that MgO strongly inhibited the growth of SRB and their H_2_S production in a dose-dependent manner (Figure 3A,B). These results are consistent with previous reports that have indicated antibacterial characteristics of various forms of magnesium [53,54].

Psychological stress has long been known to cause disruption of the gut microbiome, leading to dysbiosis [1,55]. Exposure to chronic stress in mice has shown to induce changes in the composition of the microbiota associated with both anxiety-like and depressive-like behaviors [10,56,57]. In this study, we used water avoidance stress as a well-characterized animal model of psychological stress [40,41]. Since MgO was effective in suppressing the growth of SRB in vitro, we used it as an experimental tool to test the role of SRB in WAS-induced change in host behavior. Mice exposed to WAS had increased anxiety-like behavior (Figure 4A,B; Table 1 and Table 2) compared with the control groups. However, in animals fed the MgO-enriched diet at 100 mg/kg_bw_, the anxiety-like behavior was similar to the controls. We also showed that dysbiosis may result in the overgrowth of sulfate-reducing bacteria (commensal bacteria that are typically found at very low concentrations in a healthy gut) (Figure 5).

To determine the impact of stress on SRB overgrowth in the small intestines of mice, we used 16S sequencing to quantify SRB concentrations in the mouse jejunum. We found that WAS-exposed mice had significantly more SRB in the form of Deferribacterota in the jejunum compared with the control groups (Figure 5, Table 3). Deferribacterota is a phylum of sulfate-reducing bacteria that can be found within the mouse microbiome [58], and increases in Deferribacterota have been associated with mouse models of diarrhea [59]. When MgO was added to the diet, we observed a dose-dependent reduction in stress-induced bacterial growth, with a lower concentration of SRB in the mid-third section of the mouse jejunum when the diet was supplemented with a higher dose of magnesium at 100 mg/kg_bw_ (Figure 5; Table 3). MgO also protected the animal from stress-induced anxiety-like behavior (Figure 4A,B). These findings provided support for the idea that stress changed mouse behavior, in part, because SRB overgrowth could be prevented with the addition of MgO to the diet via its suppressive effect on SRB growth and H_2_S production. These findings point to the consequences of stress on a resident gut bacterium and the effect in modifying the gut microbiota–brain axis with MgO as a possible treatment for reversing these effects.

Crosstalk between the mammalian brain and the gut microbiome occurs via a myriad of routes of communication that are still being uncovered. Gut microbes have been shown to influence the brain and behavior of their hosts by activating the immune system by acting directly on brain neurons via bacterial products [60], or by endocrine, neuronal, and immunological pathways [61]. It is also possible for pathogenic bacteria to change psychoneurological activity even without a robust immune response [14], as mice infected with *Camplybacter jejuni* [13] or *Citrobacter rodentium* [62] showed increased c-FOS immunoreactivity in the vagal sensory neurons without a rise in the concentration of circulating cytokines.

**Figure 6:**
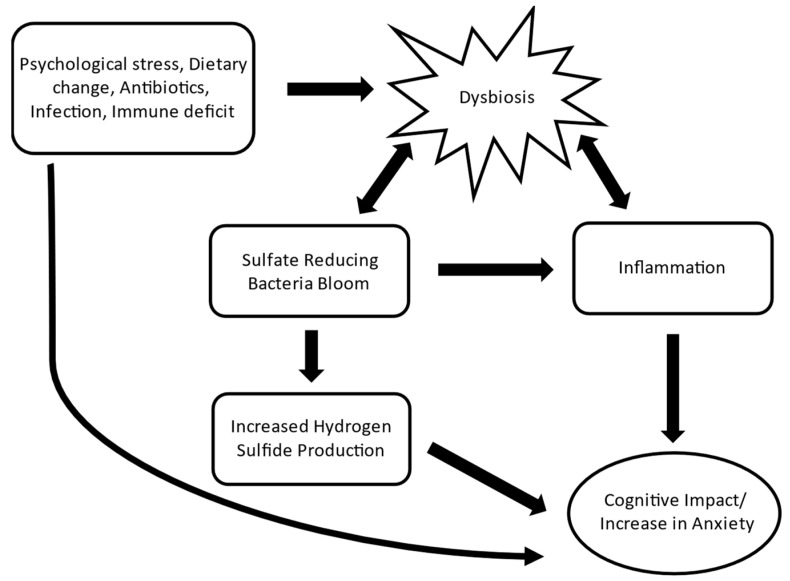
Schematic depicting how SRB blooms driven by dysbiosis can lead to increases in anxiety-like behavior. This outline shows two potential mechanisms in which dysbiosis and SRB blooms can impact behavioral and cognitive function. The first mechanism involves dysbiosis-driven inflammation. Inflammation can drive immune activation and result in cognition impairment and increased anxiety-like behavior. The second mechanism supports our hypothesis of how SRB can drive these behavioral changes through microbial by products. When dysbiosis results in SRB blooms, particularly in the small intestines, this can lead to increases in hydrogen sulfide (H_2_S) production. The small intestine is not equipped like the colon for detoxifying high levels of H_2_S, which can lead to increased exposure levels to the host. H_2_S is a known gaseous neurotransmitter, and this could potentially activate neuronal pathways resulting in impacted cognition and increased anxiety-like behavior.

The small intestine is not equipped for detoxifying high concentrations of H_2_S brought on by SRB blooms [63]. Abnormal exposure to H_2_S may therefore be an important consequence of small intestinal bacterial overgrowth where the site of fermentation and gas production expands from the large to the small intestine. The results from this study indicate a potential impact of small intestinal SRB on anxiety-like behavior. However, the underlying mechanism by which SRB impacts behavior is not known. A possible mechanism for SRB-induced behavioral change involves H_2_S (Figure 6). Hydrogen sulfide is a product of the conversion by SRB of hydrogen generated by hydrogen-producing microbes from fermentation, using sulfate as a terminal electron acceptor. Since H_2_S is also an endogenously produced neurotransmitter that participates in numerous physiologic processes, typically at low concentrations, exposure to high concentrations of this gas may adversely affect physiology [64]. The concentration of H_2_S in the colon can reach up to 1000 ppm, but the colonic mucosa is able to efficiently detoxify this gas through methylation [65]. When this gas overwhelms the limited detoxification capacity of the small intestine, abnormal exposure to high concentrations of this gas may occur. As exposure to high concentrations of H_2_S are toxic [19,66] and are associated with memory impairment and depression [67], this bacterial by product coming from the small intestine may alter behavior.

## 5. Conclusions

Previous work on SRB blooms in in vivo models are limited to correlation studies and few definitive data exist that show how SRB may impact the host and the mechanisms involved. This is the first work demonstrating that SRB can increase anxiety-like behavior in mice. Additionally, our work has shown that stress is capable of inducing the overgrowth of sulfate-reducing bacteria and anxiety-like behavior. Magnesium oxide is inhibitory on the growth of SRB and production of H_2_S and may prevent overgrowth of SRB and anxiety-like behavior in response to stress. Our data support the conclusion that stress-induced SRB blooms may induce anxiety-like behavior in a magnesium-reversible fashion in mice. In humans, SRB blooms are commonly associated with various gastrointestinal diseases [15,16]. The findings from our work indicate that SRB could potentially be responsible for behavioral changes experienced by individuals with dysbiosis-induced SRB blooms. Additionally, MgO supplementation may be beneficial in human use by helping control SRB concentrations in the gut. Future research aims to conduct clinical trials using MgO as a treatment/preventative for dysbiosis-induced SRB blooms in humans.

## Figures and Tables

**Table 1:** Latency to first exit the edge zone: mean and standard error (SEM) are reported in seconds, and a two-sided *p*-value (with a Sidak correction), for comparing the mean time of each group with the mean time for the control.

	Control	100 mg/Kg	WAS + 0 mg/Kg	WAS + 10 mg/Kg	WAS + 100 mg/Kg
Mean	5.112	5.963	9.643	10.325	6.629
SEM	0.973	1.491	1.88	1.772	1.488
*p*-value	NA	0.984	0.222	0.1	0.881

**Table 2:** Latency to first enter the center zone: mean and standard error (SEM) are reported in seconds, and a two-sided *p*-value (with a Sidak correction), for comparing the mean time of each group to the mean time for the control.

	Control	100 mg/Kg	WAS + 0 mg/Kg	WAS + 10 mg/Kg	WAS + 100 mg/Kg
Mean	21.286	17.275	44.871	30.171	21.3
SEM	7.913	3.439	12.965	5.313	3.509
*p*-value	NA	0.986	0.482	0.845	1

**Table 3:** SRB concentrations in the mouse jejunum: SRB values are represented as percentage and are multiplied by 1000 for better readability. Mean and lower/upper bounds of a 95% CI are reported, and a two-sided *p*-value (with a Sidak correction), for comparing the mean of each group with the control group mean. To account for the heavy skew of the sequencing data, the statistical analysis is performed using a shifted-log transformation of the data (with offset 1.76 × 10^−5^) so that the usual normality assumption is approximately satisfied. In Table 3, the mean concentration of each group is compared with the control group using a two-sample *T*-test with a Sidak correction to account for multiple comparisons.

	Control	100 mg/Kg	WAS + 0 mg/Kg	WAS + 10 mg/Kg	WAS + 100 mg/Kg
Mean	0.192	2.622	2.813	8.295	2.100
LB	0.071	0.590	1.178	4.105	0.446
UB	0.508	13.486	9.019	15.155	10.027
*p*-value	NA	0.040	0.001	<0.001	0.085

## Data Availability

The raw data supporting the conclusions of this article will be made available by the authors on request.
